# Hemojuvelin Predicts Acute Kidney Injury and Poor Outcomes Following Cardiac Surgery

**DOI:** 10.1038/s41598-018-20212-8

**Published:** 2018-01-31

**Authors:** Sheng-Wen Ko, Nai-Hsin Chi, Che-Hsiung Wu, Tao-Min Huang, Shih-Chieh Jeff Chueh, Chih-Hsien Wang, Jui-Hsiang Lin, Wei-Jie Wang, Jui-Tsung Ting, Huang-Ming Chang, Rory Connolly, Chien-Heng Lai, Li-Jung Tseng, Vin-Cent Wu, Tzong-Shinn Chu

**Affiliations:** 1 Division of Nephrology, Department of Internal Medicine, Taoyuan General Hospital, Ministry of Health and Welfare, Taoyuan, Taiwan; 2 Department of Surgery, National Taiwan University Hospital, Taipei, Taiwan; 3 Division of Nephrology, Department of Internal Medicine, Buddhist Tzu Chi General Hospital, Taipei Branch, Taiwan; 4 Department of Internal Medicine, National Taiwan University Hospital, Taipei, Taiwan; 5 Cleveland Clinic Glickman Urological and Kidney Institute and Lerner College of Medicine, Cleveland, OH USA; 6 National Taiwan University Hospital, Taipei, Taiwan; 7 NSARF group (National Taiwan University Hospital Study Group of ARF), Taipei, Taiwan; 8 EKF Diagnostics, Cardiff, Wales UK

## Abstract

Acute kidney injury (AKI) is detrimental after cardiac surgery. In this multicenter study, the novel biomarker hemojuvelin (HJV) was evaluated for AKI prediction following cardiac surgery. Urinary HJV, neutrophil gelatinase-associated lipocalin (NGAL), and urinary creatinine were measured in 151 patients after surgery. The outcomes of advanced AKI (KDIGO stages 2 and 3) and all causes of in-hospital mortality as the composite outcome were recorded. Areas under the receiver operator characteristic curves (AUC) and a multivariate generalized additive model (GAM) were applied to predict these outcomes of interest. Urinary HJV differentiated patients with/without AKI, advanced AKI or composite outcome after surgery (*p* < 0.001, by a generalized estimating equation) in this study. At three hours post-surgery, urinary HJV predicted advanced AKI (*p* < 0.001) and composite outcome (*p* < 0.001) with corres*p*onding AUC values of 0.768 and 0.828, respectively. The performance of creatinine-adjusted HJV was also superior to NGAL in predicting advanced AKI (AUC = 0.784 and 0.694; *p* = 0.037) and composite outcome (AUC = 0.842 and 0.676; *p* = 0.002). The integration of HJV into the Cleveland Clinic score for advanced AKI led to a significant increase in risk stratification (net reclassification improvement [NRI] = 0.598; *p* < 0.001).

## Introduction

Acute kidney injury (AKI) is a severe complication of cardiac surgery (CS), with CS being the most common cause of AKI in the surgical intensive care unit (ICU)^1^. Acute kidney injury also results in prolonged hospital stays and high mortality^2–4^. Accurate prediction and early detection of AKI is helpful in clinical decision-making and timely management.

Serum creatinine has traditionally served as the gold standard for monitoring of renal function, despite noted limitations of serum creatinine as a diagnostic marker of AKI^5^. New biomarkers were warranted to overcome these limitations, and neutrophil gelatinase-associated lipocalin (NGAL) was first studied more than a decade ago^6^. Neutrophil gelatinase-associated lipocalin has high diagnostic accuracy in the early detection of CS associated-AKI (CSA-AKI)^7^. However, some studies have reported that the performance of NGAL in predicting AKI is limited^8^ or only modest^9,10^.

Iron plays a significant role in AKI and has been a target for therapeutic intervention. However, effective therapies of AKI in animal models have not been successful in human beings. Free iron has been recognized to play an essential role in kidney injury because of its ability to catalyze the generation and propagation of reactive oxygen species (ROS)^11^ and to induce renal tubular injury via the generation of the hydroxyl radical or a similar oxidant^12^. Several proteins involved in iron metabolism in the kidney have been investigated as novel markers of kidney injury, including NGAL and hepcidin^13–15^. Hemojuvelin (HJV) is the key regulator of hepcidin expression^16,17^ and plays a crucial role in iron metabolism. Two forms of HJV, a 50 kDa membrane-bound form (mHJV) and a 42 kDa soluble form (sHJV), work in complementary roles in response to iron status^18^. The membrane-bound form is associated with decreasing total kidney iron, enhancing hepcidin secretion and promoting ferroportin internalization, whereas sHJV does the opposite^19^. Soluble HJV is extensively increased in proximal tubules of the kidney in response to ischemic and rhabdomyolysis-related AKI and is found in the urine of AKI patients after cardiac surgery^19^.

We have shown HJV to be a promising novel biomarker of AKI in animal study^19^. Urinary sHJV had a predictive accuracy similar to that of NGAL^20^, although its utility in stratifying AKI progression requires further study. In this study, HJV was validated prospectively as a urinary biomarker for AKI and mortality prediction.

## Materials and Methods

### Study Population

This study was conducted by investigators from the NSARF (National Taiwan University Study Group on Acute Renal Failure), using a prospectively constructed AKI database^21–25^. From August 2009 to January 2011, 151 patients who underwent cardiovascular surgery, including coronary bypass and valvular operations, were enrolled prospectively from a tertiary center in northern Taiwan and two local hospitals in central and northern Taiwan^26,27^. Patients who had undergone renal replacement therapy, were diagnosed as having AKI by Kidney Disease: Improving Global Outcomes (KDIGO) definitions before the operation, were younger than 18 years of age, had a history of nephrectomy or renal transplantation, or estimated glomerular filtration rate (eGFR) <30 ml/1.73 m^2^ at the time of ICU enrollment, were excluded from this study.

### Clinical Data Collection

Associated clinical information was obtained, including demographic data, comorbidity status, intervention procedures, perioperative condition, and disease severity. Serum creatinine levels and urine output were recorded at each time point after the operation as detailed in the study protocol. Operation-related parameters included methods, clamping time of the aorta, and cardiopulmonary bypass time. Post-operative inotropic agent use was quantified as per inotropic equivalents^22,24,28^. Lengths of hospital stay and ICU stay were also recorded. The Sequential Organ Failure Assessment (SOFA) score was performed as an evaluation of disease severity. The Cleveland Clinic Foundation Acute Renal Failure Scoring System (Cleveland Clinic score, see Supplementary Table S1) was also assessed to examine the risk of postoperative renal failure^29^. The data collection and all experimental protocols in this study were approved by the institutional research ethics review board (201409024RINB in National Taiwan University Hospital, 01-X16-059 in Buddhist Tzu Chi General Hospital, and TYGH104007 in Taoyuan General Hospital). All of the methods were carried out in accordance with the approved guidelines and relevant regulations. Written informed consent was obtained from all participants before inclusion.

### Sample Collection

Urine samples for measurement of urinary creatinine, HJV, and NGAL were collected in the ICU at 0, 3, 6, 12 and 24 hours after surgery. Other laboratory examinations were performed at admission and as indicated clinically thereafter. Serum creatinine (SCr) was used as the standard of AKI classification and did not change rapidly across the study time-points (see Supplementary Table S2 and Figure S1). Consequently, we did not measure the SCr level at every time-point, as with HJV and NGAL. The urine samples were centrifuged within one hour, and the sediments were discarded. The urine specimens were collected in separate polypropylene tubes containing sodium azide and stored at −80 °C until use. Each specimen was centrifuged at 800 g at 4 °C for 5 minutes, and the supernatant was utilized for ELISA analysis.

### Biomarker Measurements

The urinary HJV and NGAL levels were measured using a human HJV ELISA kit (USCN Life Science Inc., Wuhan, China) and a human lipocalin-2/NGAL ELISA kit (R&D Systems, Inc., Minneapolis, Minnesota, USA), respectively. The human HJV ELISA kit standard was characterized by LC-MS/MS analysis and Proteome Discoverer software (Thermo Fisher Scientific, version 1.4) (shown in supplementary file 2). The results were expressed in ng/mL. The lower limit of detection for HJV and NGAL was 0.156 and 0.2 ng/mL, respectively. Assays were completed as described by the manufacturer’s protocol, and each measurement was performed in duplicate. Urine creatinine levels were measured using the Jaffe assay, with standardization to the isotope dilution mass spectrometry (IDMS)-traceable reference.

### Outcome Definitions

The clinical endpoint was defined as the development of AKI (all stage), advanced AKI (stage 2 or 3 as specified by the KDIGO classification), with both urine output and creatinine criteria applied. AKI was defined as an increase in SCr of 0.3 mg/dl within 48 hours, an increase in SCr to over 1.5 times baseline within seven days or urine volume less than 0.5 ml/kg/h for 6 hours^30^. The baseline SCr measurement was defined as the pre-admission baseline SCr, where available, or the first SCr measurement in the emergency department^31^. The composite outcome of developing stage 2 or 3 AKI or in-hospital mortality was also evaluated. Patients were followed until the time of death or hospital discharge, whichever occurred first.

### Statistical Analyses

All analyses were performed with SPSS software, version 20 (IBM, Armonk, NY), R software, version 3.2.2 (Free Software Foundation, Inc., Boston, MA), and MedCalc Statistical Software, version 15.11.3 (MedCalc Software bvba, Ostend, Belgium; https://www.medcalc.org; 2015). The continuous data were expressed as mean ± standard deviation and compared using the two-tailed unpaired *t*-test. The categorical data were expressed as proportions, and the χ^2^ or Fisher’s exact test was used for comparison. We generated a receiver-operating characteristic (ROC) curve and calculated the area under the curve (AUC) to measure the performance of urinary HJV and NGAL. To assess the performance of the biomarkers with the clinical model, we calculated the improvement in Harrell’s C statistic, accounting for censoring in survival biomarker concentrations, and transformed the data using natural logarithms, if necessary, before individually adding the data to the clinical model.

To examine the effect of AKI on various time-dependent HJV and NGAL variables over time, we used the generalized estimating equation (GEE) method with the first-order autocorrelation to fit marginal linear regression models^28,32^. The linear regression analyses were performed with 95% confidence intervals (CI) based on robust standard error estimates to calculate the *p*-value. All the univariate significant and non-significant relevant covariates, creatinine-adjusted HJV at 3 hours, and some of their interactions, such as disease severity, were put on a selected variable list. The significance levels for entry (SLE) and stay (SLS) were conservatively set to 0.15. Then, with the aid of substantive knowledge, the best candidate final logistic regression model was identified manually by dropping the covariates with *p* > 0.05 until all regression coefficients were significantly different from zero. To reveal the effects of HJV for individual patients, a generalized additive model (GAM) (with spline) incorporating the subject-specific (longitudinal) random effects was plotted with adjustment for other clinical parameters to predict the outcomes^32–36^. Nonlinear effects of continuous covariates were explored using simple and multiple GAMs, which determine appropriate cut-off point(s) for discerning continuous covariates, if necessary, during the stepwise variable selection procedure. We defined the optimal cut-off value as when the log-odd equals to zero^37^.

Furthermore, we used net reclassification improvement (NRI) and integrated discrimination improvement (IDI) to examine the ability of HJV to more accurately stratify individuals into higher or lower risk categories (reclassification)^36^. In advanced AKI, an increase in NRI was calculated in a model with the Cleveland Clinic score and HJV, compared with the Cleveland Clinic score alone. In the composite outcome, an increase in NRI was calculated in a model with the SOFA score and HJV, compared with the SOFA score alone. We defined 0–10%, 10–30%, and >30% for the risk categories and reclassified the patients who died from all-causes or who developed advanced AKI. A *p*-value <0.05 was considered significant.

## Results

### Clinical Characteristics

A total of 151 patients who had cardiovascular surgery were enrolled in this study, with 51 patients identified as having AKI (33.8%) by KDIGO criteria. Twenty-nine patients (19.2%) had stage 1 AKI, 11 patients had stage 2 AKI, and 11 patients developed stage 3 AKI. The in-hospital mortality rate in this cohort was 11.3%. Table 1 shows baseline characteristics and operation-related information of patients with or without any stage of AKI and advanced AKI. Patients with any stage of AKI or advanced AKI had lower preoperative eGFR, hemoglobin and albumin levels and had higher inotropic equivalents. Patients with any stage of AKI or advanced AKI also had higher clinical severity as measured by the SOFA score when compared with the control group. A higher SOFA score in any stage of AKI or advanced AKI groups resulted in a higher rate of in-hospital mortality compared to the control group.

**Table 1:** Clinical characteristics of patients with/without AKI and advanced AKI.

	All	No AKI	Any AKI	p value	No AKI or stage 1 AKI	Stage 2 or 3 AKI	p value
	(n = 151)	(n = 100)	(n = 51)		(n = 129)	(n = 22)	
**Patient characteristics**							
Age	63.01 ± 13.59	62.16 ± 13.84	64.67 ± 13.06	0.285	62.66 ± 14.01	65.05 ± 13.17	0.448
Gender (male)	101 (66.9%)	65 (65.0%)	36 (70.6%)	0.490	89 (69.0%)	12 (54.5%)	0.183
BMI	24.91 ± 3.70	24.72 ± 3.51	25.29 ± 4.05	0.371	24.83 ± 3.52	25.35 ± 4.71	0.543
**Comorbidities**							
Hypertension	86 (54.1%)	52 (52.0%)	29 (56.9%)	0.571	68 (52.7%)	13 (59.1%)	0.579
Diabetes mellitus	36 (22.6%)	23 (23.0%)	12 (23.5%)	0.942	29 (22.5%)	6 (27.3%)	0.622
COPD	4 (2.5%)	2 (2.0%)	2 (3.9%)	0.487	3 (2.3%)	1 (4.5%)	0.549
Liver cirrhosis	8 (5.0%)	4 (4.0%)	1 (2.0%)	0.508	4 (3.1%)	1 (4.5%)	0.726
Congestive heart failure	20 (12.6%)	15 (15.0%)	4 (7.8%)	0.210	16 (12.4%)	3 (13.6%)	0.872
Malignancy	9 (5.7%)	5 (5.0%)	3 (5.9%)	0.819	7 (5.4%)	1 (4.5%)	0.865
**Laboratory data at admission**							
Pre-operative creatinine (mg/dL)	1.18 ± 0.35	1.10 ± 0.28	1.34 ± 0.42	<0.001*	1.15 ± 0.32	1.37 ± 0.47	0.041*
eGFR (MDRD) (ml/min/1.73 m^2^)	63.04 ± 21.45	66.46 ± 19.77	56.34 ± 23.17	0.006*	64.59 ± 20.96	53.96 ± 22.53	0.031*
Hemoglobin (g/dL)	12.79 ± 1.86	13.06 ± 1.78	12.24 ± 1.91	0.010*	12.92 ± 1.80	12.01 ± 2.08	0.035*
Albumin (g/dL)	4.15 ± 0.72	4.27 ± 0.64	3.92 ± 0.82	0.009*	4.26 ± 0.61	3.51 ± 0.97	0.002*
LVEF <55%	36 (22.6%)	20 (20.0%)	14 (27.5%)	0.300	26 (20.2%)	8 (36.4%)	0.093
SOFA score	6.47 ± 3.08	5.57 ± 2.51	8.26 ± 3.34	<0.001*	6.05 ± 2.74	9.00 ± 3.86	0.003*
Cleveland score	3.61 ± 1.61	3.43 ± 1.48	3.94 ± 1.79	0.069	3.46 ± 1.45	4.45 ± 2.15	0.048*
**Perioperative condition**							
Inotropic equivalents	5.63 ± 6.96	4.10 ± 4.56	8.60 ± 9.48	0.002*	4.49 ± 4.79	12.26 ± 12.27	0.008*
Presence of CPB	97 (61.0%)	54 (54.0%)	37 (72.5%)	0.028*	77 (59.7%)	14 (63.6%)	0.727
CPB time (min)	101.66 ± 138.17	78.69 ± 99.43	146.69 ± 185.94	0.017*	91.72 ± 101.52	159.91 ± 261.02	0.239
Presence of Crossclamp	75 (47.2%)	45 (45.0%)	26 (51.0%)	0.486	61 (47.3%)	10 (45.5%)	0.874
Clamp time (min)	52.31 ± 65.73	47.14 ± 66.43	62.45 ± 63.77	0.177	52.17 ± 65.99	53.14 ± 65.71	0.949
**Operative method and postoperative condition**							
CABG	82 (51.6%)	54 (54.0%)	24 (47.1%)	0.420	69 (53.5%)	9 (40.9%)	0.275
Valve	63 (39.6%)	38 (38.0%)	23 (45.1%)	0.401	51 (39.5%)	10 (45.5%)	0.601
Aorta	23 (14.5%)	11 (11.0%)	11 (21.6%)	0.082	17 (13.2%)	5 (22.7%)	0.241
length of admission (days)	21.08 ± 17.25	17.90 ± 12.25	27.44 ± 23.23	0.008*	19.61 ± 16.26	30.10 ± 20.65	0.036*
length of ICU admission (days)	4.53 ± 6.10	3.02 ± 1.68	7.56 ± 9.67	0.002*	3.82 ± 5.56	8.90 ± 7.48	0.007*
Mortality	19 (11.9%)	2 (2.0%)	15 (29.4%)	<0.001*	8 (6.2%)	9 (40.9%)	<0.001*

*p < 0.05.

Abbreviations: AKI, acute kidney injury; BMI, body mass index; CABG, coronary artery bypass graft; COPD, Chronic obstructive pulmonary disease; CPB, Cardiopulmonary bypass; eGFR, estimated glomerular filtration rate; ICU, intensive care unit; LVEF, Left ventricular ejection fraction; MDRD, The Modification of Diet in Renal Disease Study equation; SOFA score, The Sequential Organ Failure Assessment score.

### Model Prediction of AKI

**Figure 1:**
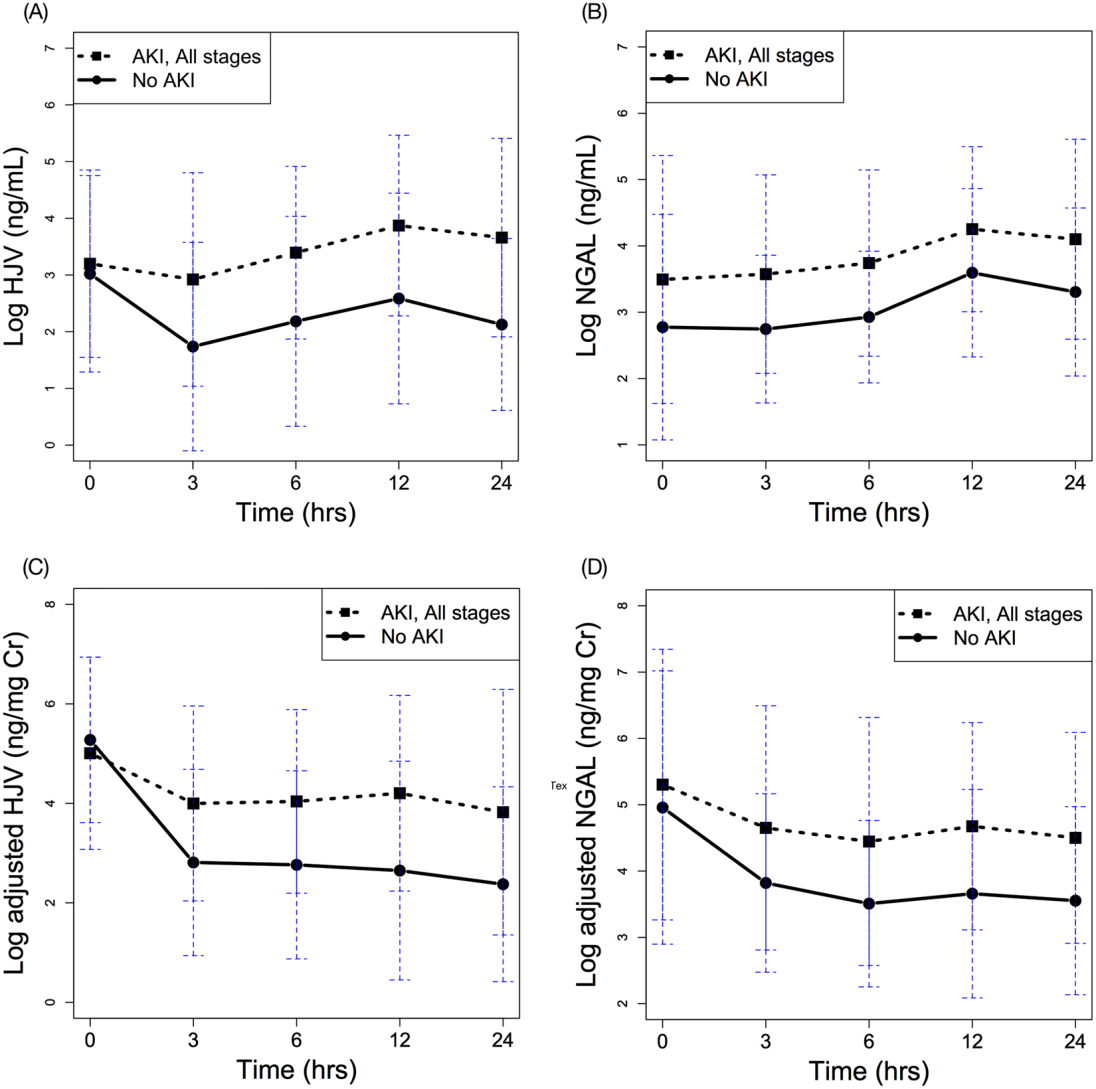
Time serial plots of post-operative HJV and NGAL biomarker levels over time, separated by patients with and without AKI. (**A**) urinary HJV, (**B**) urinary NGAL, (**C**) adjusted HJV, and (**D**) adjusted NGAL. All expressed as mean ± standard deviation of mean.

Figure 1 displays the respective HJV and NGAL levels with or without normalization to urinary creatinine at various post-operative time points in the AKI and non-AKI groups. Analysis by GEE demonstrated postoperative elevation of HJV in the AKI group (over time, *p* = 0.032). The difference in NGAL level post-surgery was also statistically significant between AKI and non-AKI groups (over time, *p* = 0.018).

**Figure 2:**
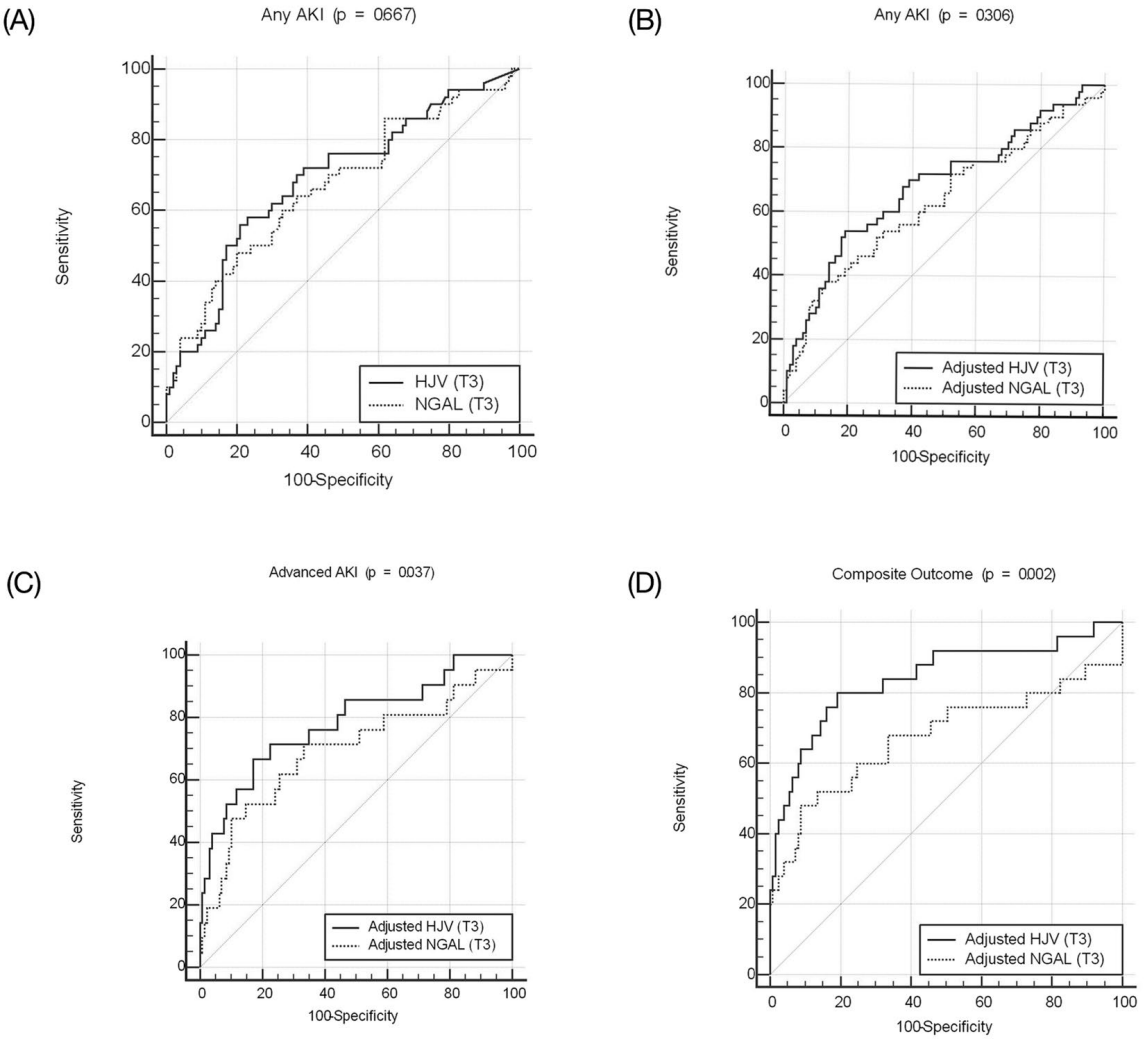
Receiver-operator characteristic curves for urinary HJV and NGAL at 3 hrs post-surgery. (**A**) HJV and NGAL, (**B**) adjusted levels predicting any stage of AKI, (**C**) adjusted levels predicting advanced AKI, and (**D**) adjusted levels predicting composite outcome.

The AUCs for HJV and NGAL at three hours post-surgery were 0.687 (95% CI 0.595 to 0.779, *p* < 0.001) and 0.666 (95% CI 0.571 to 0.761, *p* = 0.001) when predicting any stage of AKI (Table 2). After urine creatinine adjustment, the diagnostic accuracy was similar for HJV and reduced for NGAL (AUC of 0.681 and 0.632, respectively). The corresponding ROC curves for HJV and NGAL to predict AKI before (*p* = 0.667) and after (*p* = 0.306) creatinine adjustment were similar at three hours post-surgery (Fig. 2).

**Table 2:** Area under the ROC curves for AKI (all stages), advanced AKI (stage 2 or 3), or composite outcome.

AKI	T0	T3	T6	T12	T24
HJV	0.545 (0.378~0.713)	0.687 (0.595~0.779)***	0.686 (0.589~0.782)***	0.695 (0.562~0.829)**	0.733 (0.603~0.862)**
NGAL	0.633 (0.478~0.787)	0.666 (0.571~0.761)**	0.687 (0.590~0.784)***	0.658 (0.519~0.798)*	0.688 (0.543~0.833)*
adjusted HJV	0.470 (0.301~0.640)	0.681 (0.586~0.775)***	0.692 (0.594~0.789)***	0.706 (0.568~0.845)**	0.701 (0.561~0.841)**
adjusted NGAL	0.567 (0.404~0.730)	0.632 (0.533~0.730)**	0.655 (0.551~0.758)**	0.708 (0.572~0.845)**	0.687 (0.545~0.829)*
**Advanced AKI**	**T0**	**T3**	**T6**	**T12**	**T24**
HJV	0.717 (0.564~0.870)*	0.768 (0.655~0.881)***	0.748 (0.617~0.879)***	0.702 (0.548~0.857)*	0.642 (0.478~0.805)
NGAL	0.628 (0.464~0.791)	0.682 (0.526~0.828)**	0.744 (0.617~0.872)**	0.669 (0.517~0.820)*	0.678 (0.520~0.836)*
adjusted HJV	0.637 (0.475~0.800)	0.784 (0.666~0.902)***	0.847 (0.611~0.883)***	0.730 (0.583~0.878)**	0.659 (0.492~0.826)
adjusted NGAL	0.568 (0.400~0.737)	0.694 (0.553~0.834)**	0.694 (0.546~0.843)**	0.734 (0.600~0.867)**	0.726 (0.589~0.864)**
**Composite outcome**	**T0**	**T3**	**T6**	**T12**	**T24**
HJV	0.762 (0.627~0.897)**	0.828 (0.727~0.928)***	0.830 (0.727~0.933)***	0.793 (0.674~0.913)***	0.780 (0.653~0.908)***
NGAL	0.675 (0.517~0.834)*	0.673 (0.527~0.819)**	0.756 (0.641~0.871)***	0.645 (0.497~0.792)	0.653 (0.500~0.807)
adjusted HJV	0.682 (0.533~0.831)*	0.842 (0.742~0.941)***	0.831 (0.719~0.942)***	0.814 (0.702~0.926)***	0.784 (0.657~0.911)***
adjusted NGAL	0.598 (0.434~0.762)	0.676 (0.532~0.820)**	0.692 (0.551~0.832)**	0.714 (0.580~0.849)**	0.695 (0.556~0.834)*

*p < 0.05, **p < 0.01, ***p < 0.001.

Abbreviations: AKI, acute kidney injury; HJV, hemojuvelin; NGAL, neutrophil gelatinase-associated lipocalin; ROC curve, receiver-operating characteristic curve.

### Model Prediction of Advanced AKI

**Figure 3:**
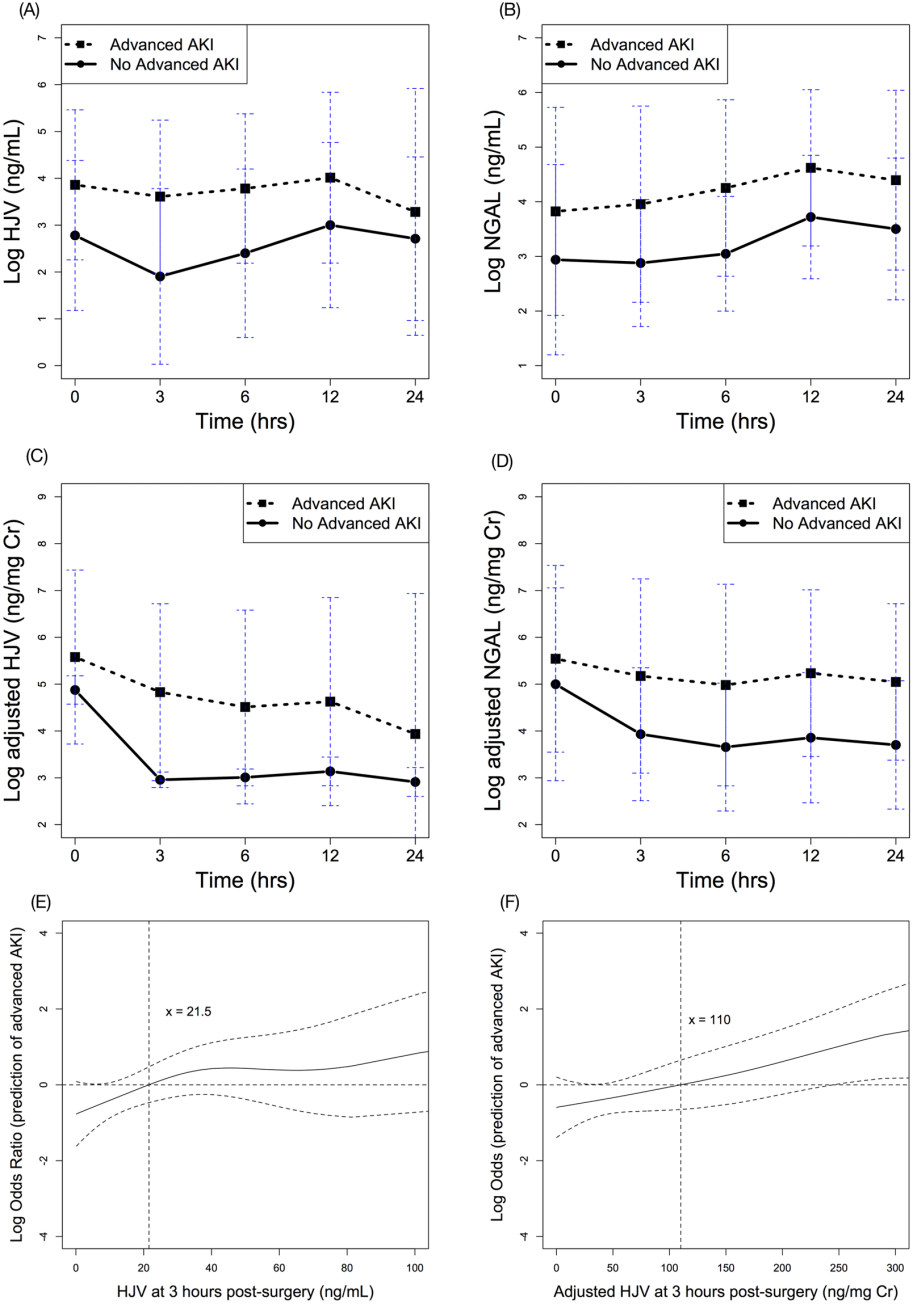
Time serial plots of post-operative HJV and NGAL biomarker levels over time, separated by patients with and without advanced AKI. (**A**) urinary HJV, (**B**) urinary NGAL, (**C**) adjusted HJV, and (**D**) adjusted NGAL. All expressed as mean ± standard deviation of mean. GAM plot for the probability of advanced AKI with HJV (**E**) and adjusted HJV (**F**) levels at 3 hours post-surgery, incorporating the subject-specific (longitudinal) random effects expressed as the logarithm of the odd (logit). The probability of outcome events was constructed with HJV levels and centered to have an average of zero over the range of the data. HJV = 21.5 ng/mL or adjusted HJV = 110 ng/mg creatinine was an independent factor for predicting postoperative advanced AKI.

The respective postoperative HJV and NGAL levels are shown in Fig. 3, with and without normalization to urinary creatinine and categorized as with or without advanced AKI. The elevation of postoperative HJV levels was demonstrated by GEE analysis in the advanced AKI group (over time, *p* = 0.040). Postoperative NGAL levels were also elevated in the GEE analysis of the advanced AKI group (over time, *p* = 0.049).

Table 2 outlines the AUCs for ROC curves generated for HJV and NGAL and the outcomes of interest. The AUCs for HJV and NGAL at three hours post-surgery were 0.768 and 0.682, respectively. With adjustment for urinary creatinine, the diagnostic accuracy of HJV and NGAL increased (AUC of 0.784 and 0.694, respectively). In our data, HJV had better diagnostic accuracy than NGAL both for those with and without advanced AKI after urinary creatinine adjustment (by AUC comparison, *p* = 0.037) (Fig. 2).

In the multivariable risk model, the independent risk factors for stage 2 or 3 AKI were a creatinine-adjusted urinary HJV level at three hours post-surgery, inotropic equivalent, and hemoglobin level (Table 3). A GAM plot was generated and showed a positive correlation between increased urinary HJV levels (with or without urinary creatinine adjustment) at three hours post-surgery and the risk of developing advanced AKI (Fig. 3). After adjusting for nonlinear effects of the variables listed in Table 1, a HJV level of 21.5 ng/mL independently predicted advanced postoperative AKI. The corresponding sensitivity and specificity was 76.2% and 69.8%, with positive and negative predictive values (PPV and NPV) of 29.1% and 94.7%, respectively. Urinary creatinine-adjusted HJV further increased the diagnostic performance, and a cut-off value of 110 ng/mg creatinine was demonstrated to predict advanced AKI. The corresponding sensitivity and specificity was 57.1% and 83.7%, with a PPV and NPV of 36.4% and 92.3%, respectively.

**Table 3:** Multivariable risk model for advanced AKI or composite outcome.

Independent variables	For advanced AKI			For composite outcome		
	Odds Ratio	95% CI	p	Odds Ratio	95% CI	p
Creatinine-adjusted HJV at 3 hours	1.303	1.023–1.659	0.032	1.926	1.131–3.282	0.016
Inotropic Equivalent	1.113	1.009–1.226	0.032	1.13	1.001–1.275	0.048
Hemoglobin	0.647	0.439–0.954	0.028	—	—	—
Albumin	—	—	—	0.211	0.069–0.649	0.007

Abbreviations: AKI, acute kidney injury; CI, confidence interval; HJV, hemojuvelin.

### Model Prediction of Composite Outcome or In-Hospital Mortality

**Figure 4:**
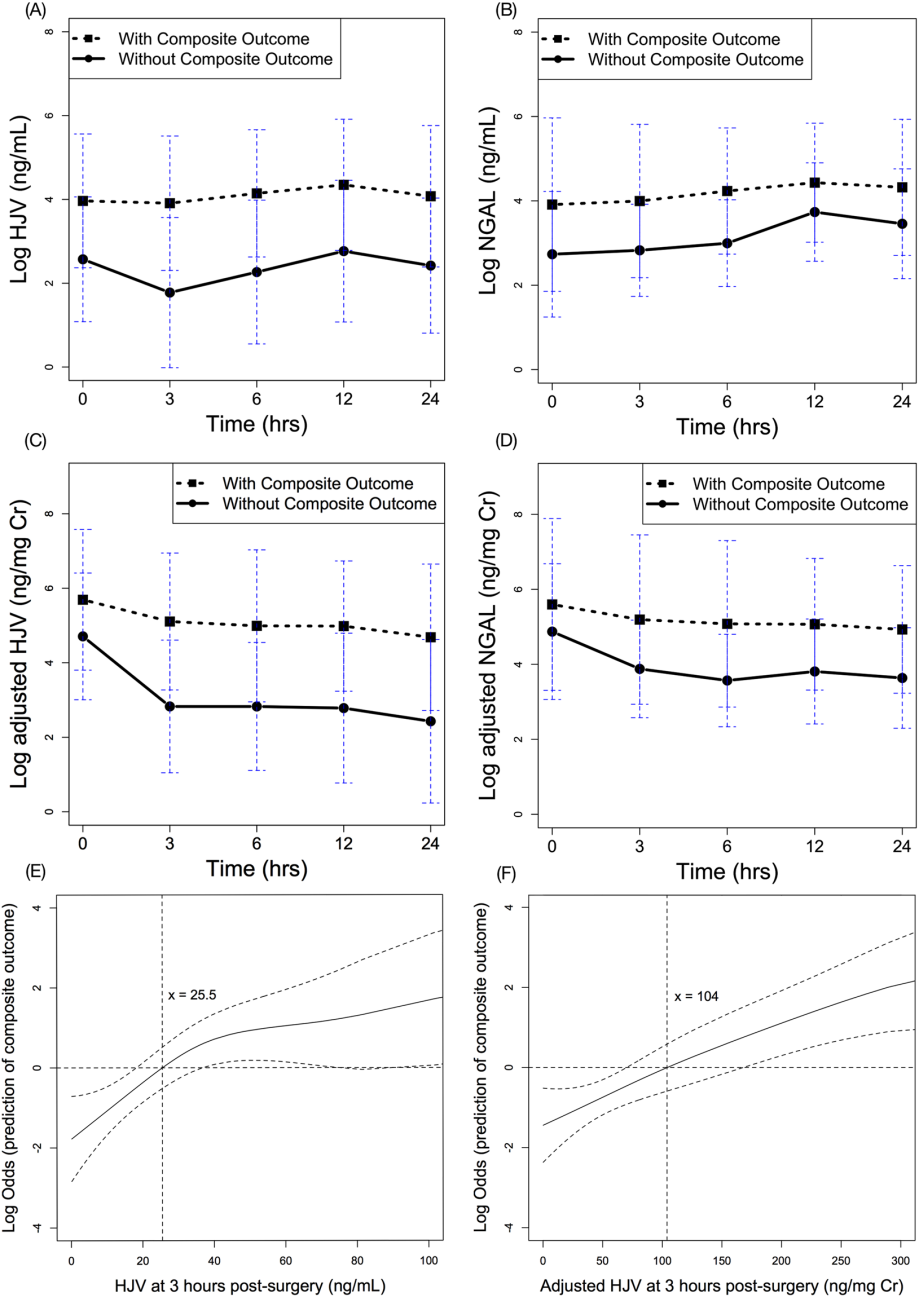
Time serial plots of post-operative HJV and NGAL biomarker levels over time, separated by patients with or without composite outcome. (**A**) urinary HJV, (**B**) urinary NGAL, (**C**) adjusted HJV, and (**D**) adjusted NGAL. All expressed as mean ± standard deviation of mean. GAM plot for the probability of composite outcome with HJV (**E**) and adjusted (**F**) levels at 3 hours post-surgery, incorporating the subject-specific (longitudinal) random effects expressed as the logarithm of the odd (logit). The probability of outcome events was constructed with HJV levels and centered to have an average of zero over the range of the data. HJV = 25.5 ng/mL or adjusted HJV = 104 ng/mg creatinine was an independent factor for predicting composite outcome. Abbreviations: AKI, acute kidney injury; HJV, hemojuvelin; NGAL, neutrophil gelatinase-associated lipocalin.

The HJV and NGAL levels differed significantly in the two aforementioned groups by the GEE model analysis predicting the composite outcome, with *p* < 0.001 and p = 0.048, respectively (Fig. 4). The AUCs values for the ROC curves (Table 2) had substantial predictive power for HJV levels at all time-points, with the highest values at three hours (AUC 0.828, *p* < 0.001) and six hours (AUC 0.830, *p* < 0.001) post-surgery.

The NGAL levels also notably predicted the composite outcome before six hours, with the highest AUC at six hours post-surgery (0.756, *p* < 0.001). Adjustment for urinary creatinine further improved the diagnostic accuracy of HJV at three and six hours post-surgery (AUC of 0.842 and 0.831, respectively). HJV had better diagnostic accuracy than NGAL for the composite outcome (by AUC comparison, *p* = 0.004), especially after urinary creatinine adjustment (by AUC comparison, *p* = 0.002) (Fig. 2).

The multivariable model generated for predicting the composite outcome by creatinine-adjusted HJV is shown in Table 3. Independent variables were applied to generate the GAM graph (Fig. 4), in which HJV levels at three hours post-surgery were plotted against the log of the odds of the probability of composite outcome. The cut-off value in the GAM model, i.e., HJV = 25.5 ng/mL, translated into a sensitivity of 84.0% and a specificity of 76.0%, while the PPV and NPV was 41.2% and 95.0%, respectively. After adjustment for urinary creatinine, 104 ng/mg creatinine was the cut-off value to predict the postoperative composite outcome. The corresponding sensitivity and specificity was 68.0% and 86.4%, with a PPV and NPV of 50.0% and 93.1%, respectively.

### Addition of Creatinine-Adjusted HJV to the Cleveland Clinic Score and SOFA Score

We examined reclassification for detection of advanced AKI through the addition of creatinine-adjusted HJV to the Cleveland Clinic score. This led to a significant increase in risk stratification (total NRI = 0.598; 95% CI, 0.287 to 0.908; *p* < 0.001). The majority of this increase was observed for advanced AKI events (NRI event = 0.35; 95% CI, 0.063 to 0.637; *p* = 0.017), whereas the NRI non-event was 0.248 (95% CI, 0.128 to 0.368; *p* < 0.001). Similarly, the total IDI was significant at 0.199 (95% CI, 0.093 to 0.305; *p* < 0.001).

We also analyzed reclassification for detection of composite outcome. The addition of creatinine-adjusted HJV to the SOFA score led to a notable increase in risk stratification (total NRI = 0.567; 95% CI, 0.269 to 0.864; *p* < 0.001). The majority of this increase was observed for composite outcome events (NRI event = 0.36; 95% CI, 0.091 to 0.629; *p* = 0.009), whereas the NRI non-event was 0.207 (95% CI, 0.079 to 0.334; *p* = 0.001). Correspondingly, the IDI was also significant at 0.183 (95% CI, 0.081 to 0.285; *p* < 0.001).

## Discussion

In this study, HJV was validated as a novel urinary biomarker for AKI prediction. To our knowledge, this is the first study to use HJV as an AKI biomarker and to compare its predictive power with NGAL. The creatinine-adjusted HJV level at 3 hours post-surgery predicted the occurrence of AKI, particularly advanced AKI and/or in-hospital mortality, while also showing a better predictive power than NGAL. The addition of urinary HJV to the Cleveland Clinic score and the SOFA score led to a significant improvement in outcome stratification.

HJV, a glycophosphatidylinositol (GPI)-linked membrane protein, is highly expressed in liver and skeletal muscle. Multiple studies have shown that iron plays a major role in ROS-induced nephrotoxicity^19,38^. The interaction of hepatocyte HJV with bone morphogenic protein 6 (BMP6), matriptase-2, and neogenin plays a critical role in hepcidin expression and systemic iron regulation^39–41^. Membrane-bound HJV is cleaved by furin, a proprotein convertase, and the cleaved HJV is released from the cells in soluble form (sHJV). There is evidence that the increased expression of the HJV-hepcidin-ferroportin pathway is an intrinsic response in the kidney under iron overload conditions during AKI^19^, and that the release of sHJV is a local tissue-specific mechanism, signaling the local iron requirements of hypoxic kidneys independent of the oxygen status of the liver or muscle^42^. Kidney biopsy results from humans and animals with AKI have also showed increased HJV staining in renal proximal tubules^19^. While analyzing advanced AKI in our data, the significant predictive value was obtained using the urinary HJV biomarker at three hours post-surgery. This finding reinforced our previous study, in which high levels of urinary sHJV were observed in patients after cardiac surgery and rhabdomyolysis-related AKI^19^.

To our knowledge, this is the first time urine sHJV levels have been examined in an AKI patient cohort. There was no clinical data available for the actual fractional excretion of sHJV, and elevation of urinary sHJV may be taken from filtration or secretion. In a recent study among CKD patients, there was no association between sHJV and eGFR, suggesting that factors other than the renal elimination of the peptide are responsible for high serum sHJV levels^43^. Likewise, kidney biopsies from both human AKI and ischemia-reperfusion injured mice showed high expression of HJV in the proximal renal tubule. However, the expression in the glomerulus is scared. In light of this, HJV expression was localized to the injured tubules with obvious features of cast formation, tubular dilation, and tubular necrosis under the most intense hematoxylin and eosin (H&E) staining^19^.

Neutrophil gelatinase-associated lipocalin is an iron metabolism regulator and delivers iron in and out of cells^44,45^. Neutrophil gelatinase-associated lipocalin was previously found to be augmented by sHJV and down-regulated by furin inhibitor both *in vitro* and in an animal study^19^, which implies that HJV is closely associated with NGAL in kidney injury. In an animal ischemia-reperfusion model, Western blot analysis showed that the sHJV rapidly increased at three hours, whereas NGAL increased later at 3–24 hours^19^. It also demonstrated that sHJV expression was correlated with, and faster than, the expression of NGAL in the mouse kidney. In advanced AKI, more significant AUCs were obtained because extensive tubular damage in the renal tubule may have further amplified the difference in HJV expression compared to milder forms of AKI or those cases without clinical kidney injury. This supports the view that HJV is not only a suitable marker for early prediction of AKI but that it is also helpful for stratifying disease progression, identifying high-risk AKI patients, and making clinical decisions such as dialysis initiation.

In clinical practice, a prognostic or prediction model can forecast individual patient risk and predict hospital mortality of critically ill patients before administration of acute dialysis. We are desperate for markers to determine which patients will develop advanced AKI and if the disease will progress to a serious outcome. The addition of HJV to a clinical model resulted in greater IDI and NRI and was shown to improve risk prediction. These results demonstrated that addition of creatinine-adjusted HJV to the Cleveland Clinic score could improve the accuracy of predicting advanced AKI and its addition to the SOFA score could also enhance the ability to predict advanced AKI and death. These results may be useful in early recognition of patients with a higher risk of severe outcomes and in reducing the uncertainty of daily clinical decision-making.

Generally, the goal for adjustment of urinary compounds with the creatinine level is to extrapolate the value to a 24-hour excretion amount. However, the serum creatinine level and urinary creatinine excretion are not under steady state during AKI. This is further complicated by impaired creatinine secretion after renal tubular damage. Therefore, the role of creatinine adjustment for urinary biomarkers is uncertain^46^. Ralib *et al*. found normalization with urinary creatinine concentration improves the prediction of developing AKI, but not established AKI^47^. Waiker *et al*. suggested that the collection of timed urine specimens to estimate the actual excretion rate is the most accurate method to quantify biomarkers^48^. In our data, adjustment with urinary creatinine concentration increased the diagnostic power of HJV (in AUC analyses), and we provided values both before and after urinary creatinine adjustment, for clinical application to predict advanced AKI and composite outcome.

In our multivariate logistic regression analysis, we found that adjusted urinary HJV levels at three hours post-surgery and inotropic equivalent were independent risk factors for both advanced AKI and the composite outcome. Through this multivariate model, we determined the best cut-off values for unadjusted and adjusted HJV to predict advanced AKI and composite outcome for real-world practice. Low serum albumin level was also an independent risk factor for composite outcome. Albumin was reported to improve renoperfusion either by prolonging renal vasodilation through reaction with oxides of nitrogen^49^ or by facilitating fluid transport in the medulla in peritubular capillaries^50^.

The strengths of this study included the multi-center design and homogenous (post-cardiovascular surgery) patient population. Furthermore, we compared HJV to the well-known novel AKI biomarker, NGAL, and demonstrated HJV had superior diagnostic power. Membrane-bound HJV is associated with reducing iron content in the kidney, hepcidin secretion, and ferroportin degradation in AKI. As a result, we raised the proposition that increasing the expression levels of membrane-bound HJV may be a potential therapeutic option for AKI. Approaches to target iron trafficking via HJV may offer new insights. However, there were some limitations with this study. First of all, although the homogenous patient population was satisfactory for comparison and analysis, this result could not represent all AKI patients from other known causes, such as sepsis or drug-induced AKI. Therefore, larger prospective studies are required to validate HJV for different etiologies of AKI. Secondly, the interpretation of urinary HJV is not clear in AKI patients with underlying chronic kidney disease (CKD). Limited evidence displayed an increased serum HJV level in patients receiving hemodialysis, but this was not shown in CKD patients without dialysis or patients that received a kidney transplant^43^. Further studies to confirm the predictive power of HJV in CKD patients are warranted.

## Conclusion

Our data demonstrated that HJV might be a useful marker for the early diagnosis of AKI and advanced AKI after cardiovascular surgery, with enhanced predictive capability when compared to NGAL. HJV also improved the predictive ability of the Cleveland Clinic score for advanced AKI and the SOFA score for composite outcome. Further validation in a larger population is necessary to confirm this finding.

## Electronic supplementary material

Supplementary File
