# Supplementary material for: Hemojuvelin Predicts Acute Kidney Injury and Poor Outcomes Following Cardiac Surgery
Source: Sci Rep. 2018 Jan 31;8:1938. doi: 10.1038/s41598-018-20212-8 (PMC5792584; doi:10.1038/s41598-018-20212-8)
Supplement: Supplementary file 1 — Supplementary File [file 41598_2018_20212_MOESM1_ESM.pdf]

Supplementary file

Hemojuvelin Predicts Acute Kidney Injury and Poor Outcomes Following Cardiac Surgery

Sheng-Wen Ko, Nai-Hsin Chi, Che-Hsiung Wu, Tao-Min Huang, Shih-Chieh Jeff Chueh, Chih-Hsien Wang, Jui-Hsiang Lin, Wei-Jie Wang, Jui-Tsung Ting, Huang-Ming Chang, Rory Connolly, Chien-Heng Lai, Li-Jung Tseng, Vin-Cent Wu, Tzong-Shinn Chu

Correspondence and reprint requests to:

Vin-Cent Wu, **MD, PhD;**

Department of Medicine, National Taiwan University Hospital,

7 Chung-Shan South Road, Zhong-Zheng District, Taipei 100, Taiwan,

Email: q91421028@ntu.edu.tw

Phone: +886 2 23123456 × 63098, and fax: +886 2 23952333.

Supplementary File 1

Table S1: The Cleveland Clinic Foundation Acute Renal Failure Scoring System

Risk Factor	Points
Female gender	1
Congestive heart failure	1
Left ventricular ejection fraction < 35%	1
Chronic obstructive pulmonary disease	1
Insulin-requiring diabetes	1
Previous cardiac surgery	1
Preoperative use of IABP	2
Emergency surgery	2
Valve surgery only (reference to CABG)	1
CABG plus valve (reference to CABG)	2
Other cardiac surgeries	2
Preoperative creatinine 1.2 to < 2.1 mg/dl (reference to 1.2)	2
Preoperative creatinine ≥ 2.1 mg/dl (reference to 1.2)	5

Abbreviation: CABG, Coronary artery bypass grafting; IABP, Intra-aortic balloon pump.

Table S2:

AKI	T3	T6
adjusted HJV	0.671 (0568~0.774)	0.693 (0.592~0.794)
Cr	0.698 (0.607~0.779)	0.772 (0.686~0.844)
ROC comparison	p = 0.7149	p = 0.2628

Advanced AKI	T3	T6
adjusted HJV	0.833 (0.718~0.947)	0.808 (0.683~0.932)
Cr	0.675 (0.583~0.758)	0.622 (0.529~0.709)
ROC comparison	p = 0.0775	p = 0.0777

Composite outcome	T3	T6
adjusted HJV	0.867 (0.780~0.955)	0.857 (0.760~0.955)
Cr	0.637 (0.544~0.723)	0.623 (0.529~0.710)
ROC comparison	p = 0.0025	p = 0.0047

Figure S1:

Receiver-operator characteristic curves for urinary creatinine-adjusted HJV and serum creatinine at 3 and 6 hours post-surgery.

(A)

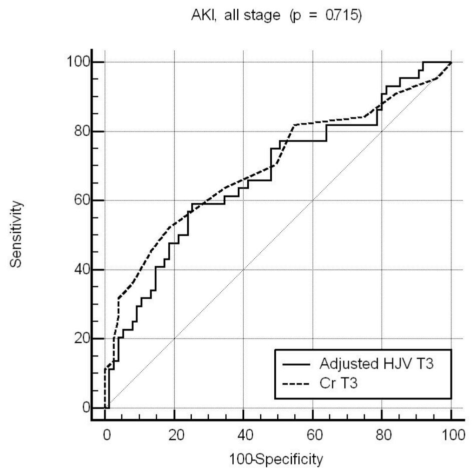

(B)

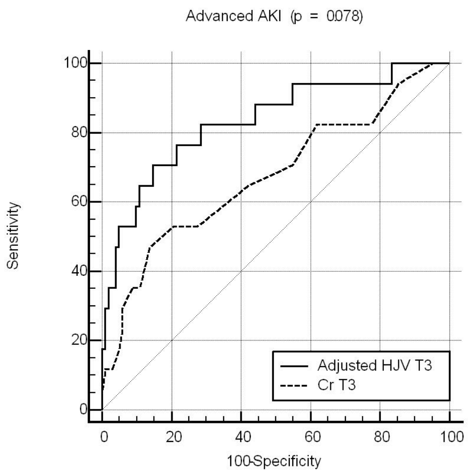

(C)

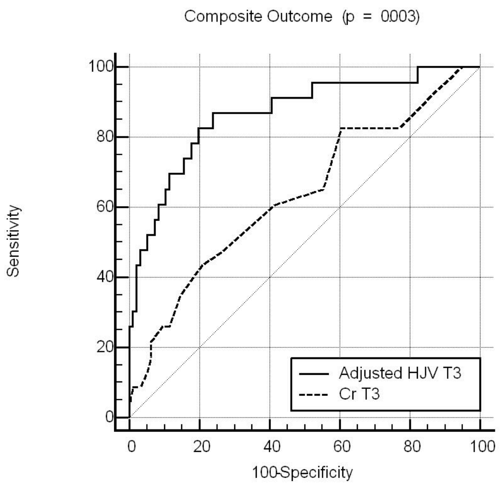

(D)

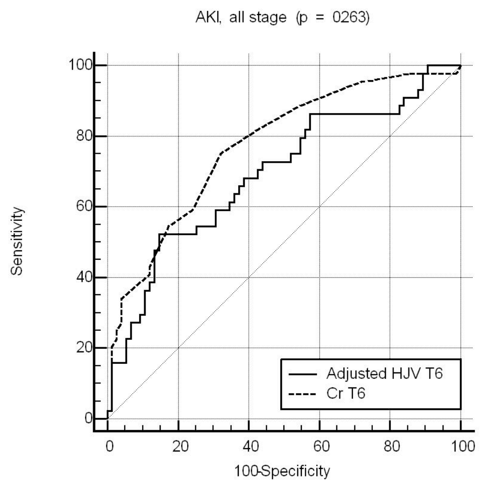

(E)

(F)

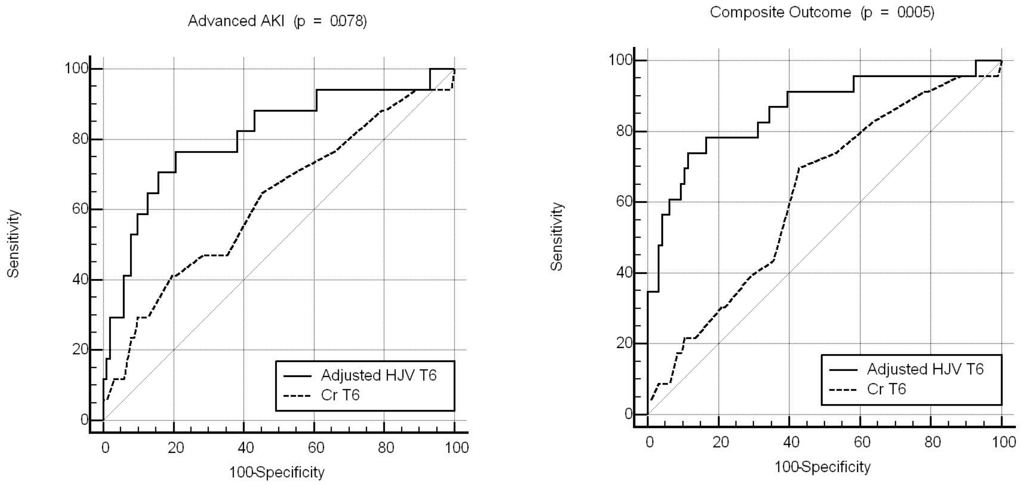

(A) predicting AKI (all stages), (B) predicting advanced AKI, and (C) predicting composite outcome at 3 hours.

(D) predicting AKI (all stages), (E) predicting advanced AKI, and (F) predicting composite outcome at 6 hours.

Result:

We have shown a comparison of the area under the receiver-operating characteristic curves between serum creatinine and urinary HJV at 3 and 6 hours. With respect to composite outcome, the performance of urinary creatinine-adjusted HJV was better than serum creatinine alone.

References:

Thakar, C. V., Arrigain, S., Worley, S., Yared, J. P. & Paganini, E. P. A clinical score to predict acute renal failure after cardiac surgery. *J Am Soc Nephrol* **16**, 162-168, doi:10.1681/ASN.2004040331 (2005).

Supplementary File 2

Validaiton of the recombinant protein standard from ELISA (USCN LIFE SCIENCE, INC)

Materials and methods

LC-MS/MS analysis performed by TOOLS BIOTECH, INC. Briefly, the digested HJV recombinant protein, which was kindly provided by USCN LIFE SCIENCE, INC was analyzed by LC-MS/MS using a Dionex Ultimate 3000 RSLCnano system. This analysis was coupled with a Hybrid mass spectrometer (Thermo Orbitrap Elite). The data analysis was carried out using Proteome Discoverer software (version 1.4, Thermo Fisher Scientific). The MS/MS spectra were searched against the SwissProt database (released on Mar 16, 2016, extracted for Homo sapiens, 20,199 sequences) using the Mascot search engine (Matrix Science, London, UK; version 2.5).

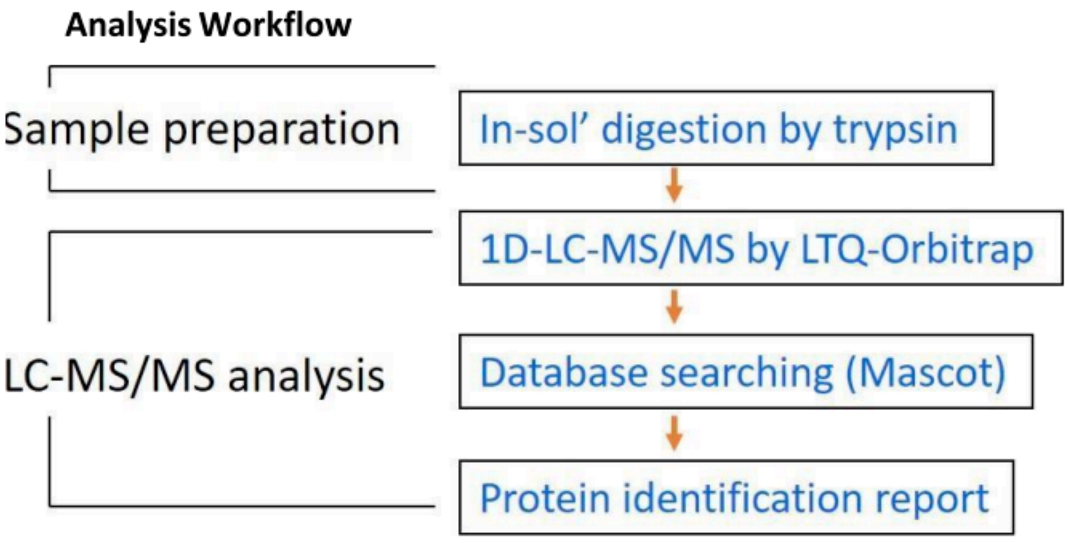

Results

This protein identification result showed 65.74% of the relative protein abundance is human hemojuvelin. This result supports that the protein indentification of our samples should be HJV.

Summary of search results

We have sucessfully identified the target protein Hemojuvelin with 6 unique peptides in C-terminal region of protein. The detail search result was showed in Data Report.

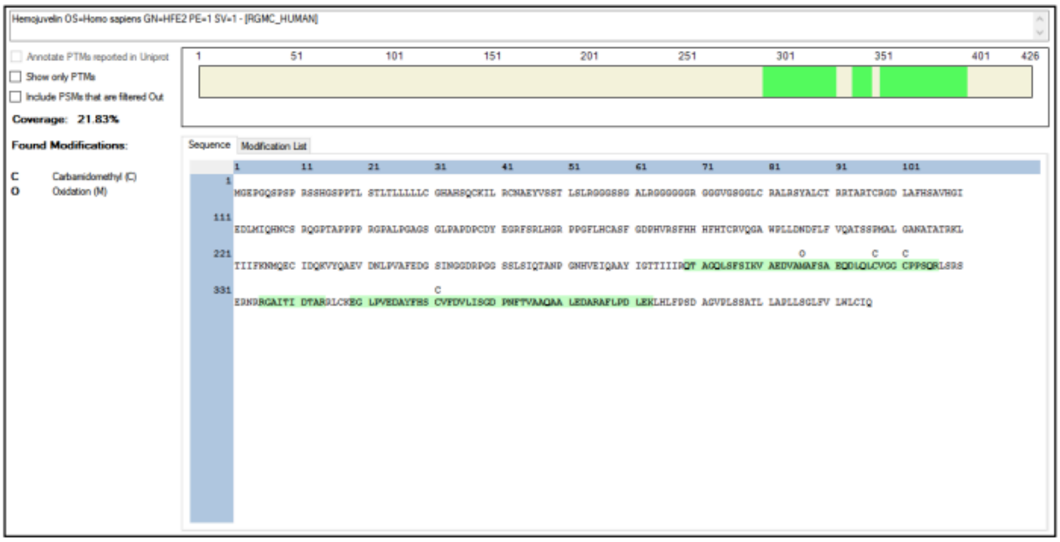

Sample	Accession	Identity	Species	# Unique peptides	Score	# AAs	MW (kDa)	Sequence coverage
RGMc_Human	Q6ZVN8	Hemojuvelin	*Homo sapiens*	6	2014.33	426	45.1	21.83%

Denoted : Hemojuvelin = Homo sapiens GN = RGMC_HUMAN
